# CaMKII in sinoatrial node physiology and dysfunction

**DOI:** 10.3389/fphar.2014.00048

**Published:** 2014-03-18

**Authors:** Yuejin Wu, Mark E. Anderson

**Affiliations:** ^1^Department of Internal Medicine, Carver College of Medicine, University of IowaIowa City, IA, USA; ^2^Department of Molecular Physiology and Biophysics, Carver College of Medicine, University of IowaIowa City, IA, USA

**Keywords:** calcium/calmodulin-dependent protein kinase II, sinoatrial node, heart rate, sinoatrial node dysfunction, calcium

## Abstract

The calcium and calmodulin-dependent protein kinase II (CaMKII) is present in sinoatrial node (SAN) pacemaker cells and is required for physiological “fight or flight” SAN beating rate responses. Inhibition of CaMKII in SAN does not affect baseline heart rate, but reduces heart rate increases in response to physiological stress. CaMKII senses intracellular calcium (Ca^2^^+^) changes, oxidation status, and hyperglycemia to phosphorylate substrates that regulate Ca^2^^+^-sensitive proteins, such as L*-*type Ca^2^^+^ channels, phospholamban, and cardiac ryanodine receptors (RyR2). All of these substrates are involved in the SAN pacemaking mechanism. Excessive CaMKII activity, as occurs under pathological conditions such as heart failure, ischemia, and diabetes, can promote intracellular Ca^2^^+^ overload and reactive oxygen species production. Oxidation of CaMKII (ox-CaMKII) locks CaMKII into a constitutively active configuration that contributes to SAN cell apoptosis and fibrosis. This ox-CaMKII-mediated loss of functional SAN cells contributes to SAN dysfunction (SND) and sudden death. Thus, CaMKII has emerged as a central regulator of physiological SAN responses and a key determinant of SND.

## INTRODUCTION

The sinoatrial node (SAN) is a specialized region of heart tissue present at the junction of the right atrium and superior vena cava that extends along the cristae terminalis, where it initiates each normal heart beat. The pacemaking function of SAN cells is accomplished by generation of spontaneous action potentials. There appear to be redundant systems in SAN for generating spontaneous cell membrane potential depolarizations, which are ultimately necessary to sustain life by maintaining cardiac output. One of these systems comprises a set of cell membrane delimited ion channels. These ion channels include hyperpolarization-activated cyclic nucleotide-gated (HCN) channels that conduct an inward current, sometimes called a pacemaker current or funny current (I_f_; DiFrancesco, 1991), L-type (Ca_V_1.2/1.3; Christel et al., 2012) and T-type (Ca_V_3.1/3.2) Ca^2^^+^ channels (Mangoni et al., 2006; Tanaka et al., 2008; Brahmajothi et al., 2010) and several K^+^ channels, including ERG (Brahmajothi et al., 1997, 2010) and KvLQT1 (Chandler et al., 2009; Brahmajothi et al., 2010 ). All of these ion channels have the potential to play a role in pacemaking under different conditions. The other system involves intracellular Ca^2^^+^ machinery that is used for excitation–contraction coupling in mechanically purposed myocardium, but that contributes to rhythmic intracellular Ca^2^^+^ oscillations in SAN. This system enables SAN fight or flight heart rate increases and contributes to SAN cell death under pathological stress. These components include the sarcoplasmic reticulum (SR; Rigg and Terrar, 1996), which contains the sarco/endoplasmic reticulum Ca^2^^+^-ATPase (SERCA2a), the ryanodine receptor 2 (RYR2), a large Ca^2^^+^ channel that releases Ca^2^^+^ from the SR lumen to the cytoplasm and the cell membrane spanning Na^+^/Ca^2^^+^ exchanger (NCX1; Sanders et al., 2006). The components in both systems collaborate but are also capable of independent activity that ensures nonstop pacemaking activity (Lakatta and DiFrancesco, 2009; Lakatta et al., 2010).

We believe that the effects of the multifunctional Ca^2^^+^ and calmodulin-dependent protein kinase II (CaMKII) on SAN cell biology are related to actions on Ca^2^^+^ homeostasis. CaMKII is a multifunctional serine/threonine-specific protein kinase that is initially activated by the Ca^2^^+^/calmodulin complex (Schulman and Greengard, 1978). CaMKII is present in contracting myocardium and in SAN cells (Vinogradova et al., 2000). Details of CaMKII structure, function, activation, and inactivation are contained in another chapter in this compendium (XYZ). However, the CaMKII holomeric structure allows it to perform as a precisely regulated enzyme that activates and inactivates with Ca^2^^+^/calmodulin binding and unbinding but also to transition into a constitutively active conformation by post-translational modifications to the autoregulatory domain (Kuret and Schulman, 1985; Erickson et al., 2008, 2013; Chao et al., 2011; Gutierrez et al., 2013). Excessive levels of constitutively active CaMKII are linked to cardiovascular and pulmonary diseases, including SAN dysfunction (SND; Erickson et al., 2011; Sanders et al., 2013).

## CaMKII IN SAN PHYSIOLOGY

Activated CaMKII can catalyze phosphorylation of multiple Ca^2^^+^ homeostatic proteins, including L-type, e.g., Ca_V_1.2 (Dzhura et al., 2000; Grueter et al., 2006) and T-type, e.g., Ca_V_3.2 (Yao et al., 2006) Ca^2^^+^ channels, phospholamban (PLN; Lindemann et al., 1983), a protein that negatively regulates SERCA2a in the absence of CaMKII or protein kinase A catalyzed phosphorylation (Kranias and Hajjar, 2012), and RYR2 (Witcher et al., 1991; Wehrens et al., 2004). CaMKII catalyzed phosphorylation increases Ca^2^^+^ entry through Ca^2^^+^ channels, increases SERCA2a uptake of cytoplasmic Ca^2^^+^ into the SR lumen through phosphorylating PLN, which in turn increases the pool of SR releasable Ca^2^^+^, increases Ca^2^^+^ release from RYR2 by phosphorylation of RYR2 at several sites, including serine 2814. On one hand, these effects will increase intracellular Ca^2^^+^ flux through the SR and RYR2 to accelerate NCX1 to increase SAN cell action potential frequency and the physiological fight or flight heart rate response. On the other hand, excessive CaMKII activity will cause Ca^2^^+^ overload (Wagner et al., 2011), which can induce increased reactive oxygen species (ROS) production and cause SAN cell damage or death (Swaminathan et al., 2011; Luo et al., 2013).

**FIGURE 1:**
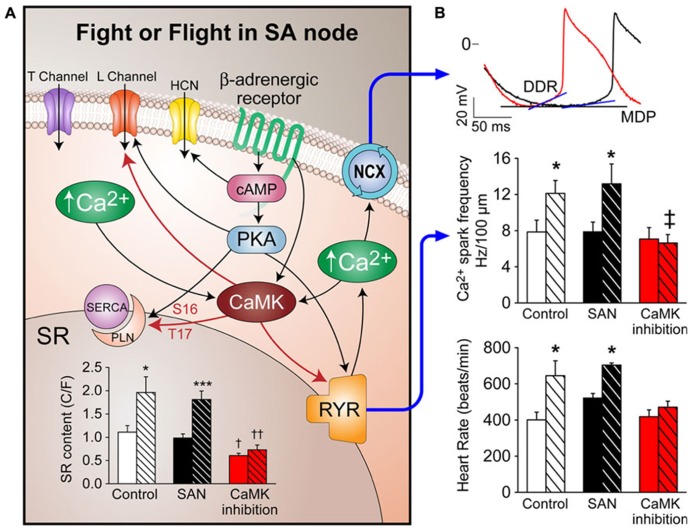
**Mechanism of CaMKII effects on Fight or Flight in SA node**. **(A)** Fight or flight stimulation (e.g., isoproterenol) activates PKA and CaMKII, which phosphorylate L-type Ca^2^^+^ channels and PLN to increase SR Ca^2^^+^ content. RYR2 phosphorylation increases Ca^2^^+^ release from SR. Increased Ca^2^^+^ release accelerates NCX1 activity which increases the diastolic depolarization rate (DDR) of SAN action potentials **(B)**. SR Ca^2^^+^ content increases by isoproterenol are abolished by CaMKII inhibition. Upper panel shows DDR change with isoproterenol (red trace) compare to control DDR (black trace), MDP, maximal diastolic potential. Middle panel shows Ca^2^^+^ spark frequency increases after isoproterenol are abolished by CaMKII inhibition. The lower panel shows the heart rate increase by isoproterenol is abolished by CaMKII inhibition. All shaded bars in bar graphs represent data with isoproterenol effects. Both white bars (WT control) and black bars (transgenic control) represent data from control SAN. *p < 0.05, ***p < 0.001 before vs. after isoproterenol, † *p* < 0.05 compare to control SAN groups, †† or ‡ *p* < 0.01 compare to control SAN groups.

The role of CaMKII in SAN function has been explored since 1989 (Hagiwara and Irisawa, 1989). The major focus of this study was on the effects of calmodulin or CaMKII on I_f_ currents by using calmidazolium, a calmodulin inhibitor with many off target actions (Klöckner and Isenberg, 1987). They found that I_f_ currents were sensitive to intracellular Ca^2^^+^ but no evidence that I_f_ was regulated by CaMKII. A more recent study (Rigg et al., 2003) confirmed that I_f_ currents are regulated by Ca^2^^+^ and calmodulin but not by the CaMKII pathway. They showed that I_f_ current amplitude was unaffected by the CaMKII inhibitor KN-93 (1 μM) although this CaMKII inhibition did reduce L-type Ca^2^^+^ current by 48 ± 19% at 0 mV voltage clamp command potential. However, a more recent study challenged the concept of calmodulin regulation of I_f_ (Chatelier et al., 2005) based on experiments in inside-out cell membrane macro-patches excised from rabbit SAN cells. They found that “intracellular” calmodulin perfusion had no effect on HCN activity and did not change the cAMP-induced I_f_ activation shift. This study suggested that another calmodulin inhibitor, W-7, with well documented off target actions had direct effects on I_f_ that were independent of Ca^2^^+^ and calmodulin. The myriad off target actions on ion channels represent major obstacles to the use of CaMKII inhibitors in functional studies ( Ledoux et al., 1999; Gao et al., 2006; Rezazadeh et al., 2006; Liao et al., 2011). CaMKII enhances Ca_V_1.2 channel currents in ventricular myocytes (Anderson et al., 1994; Xiao et al., 1994; Yuan and Bers, 1994) and so could potentially affect SAN automaticity by actions on Ca_V_1.2. A paper from the Xiao group showed that CaMKII was likely to play an important role in SAN pacemaker activity by actions at voltage-gated Ca^2^^+^ channels (Vinogradova et al., 2000). They were able to stop SAN cell automaticity by using CaMKII inhibitors KN-93 or myristoylated autocamtide-2-related inhibitory peptide (AIP) (a cell membrane permeant peptide inhibitor modeled after the CaMKII autoinhibitory region). The findings from the Xiao group supported an I_f_-independent role for cardiac pacing. However, these studies were mostly performed using small molecule inhibitors with off target actions that complicate interpretation of the results. Taken together, these findings highlight some of the limitations of available small molecule calmodulin and CaMKII antagonists and suggest that I_f_ is not directly responsive to calmodulin or CaMKII but leave open the question whether CaMKII actions at Ca_V_1.2 channels contribute to SAN automaticity. We developed a mouse with myocardial targeted transgenic expression of AC3-I, a highly selective CaMKII inhibitory peptide, under control of the α-myosin heavy chain promoter (Zhang et al., 2005). AC3-I expression was present in SAN cells and a study from our group using this mouse found that CaMKII inhibition did not affect baseline SAN pacemaking activity but selectively impaired the fight or flight response of SAN cells to isoproterenol (Wu et al., 2009). CaMKII was responsible for approximately half of the dynamic heart rate response range. We found that neither SAN cell Ca^2^^+^ channels nor I_f_ currents from AC3-I mice were different compared with their WT littermates nor control transgenic mice expressing AC3-C, an AC3-I like peptide without biological activity. We found that SR Ca^2^^+^ content responses to isoproterenol in those mice were reduced, potentially as a consequence of diminished CaMKII catalyzed phosphorylation of PLN. The reduced SR Ca^2^^+^ content likely contributed to reduced Ca^2^^+^ spark frequency as well as decreased Ca^2^^+^ release from SR (**Figure 1**). Our findings were later confirmed by studies from another group using a CaMKIIδ knock out mouse (Xu et al., 2010). Their study also showed that CaMKII is required for heart rate increases by isoproterenol stimulation or in response to a physiological fight or flight mechanism. A recent study from Terrar group (Collins and Terrar, 2012) suggested that the effect of CaMKII in atrial myocytes may be primarily on SR proteins due to different distribution of CaMKII in ventricular myocytes compare to atrial myocytes which lack of T-tube. The effects of CaMKII on atrial Ca^2^^+^ channels are indirectly through CaMKII enhanced SR Ca^2^^+^ releasee that stimulates adenylyl cyclases (ACs). Recently, one study from Lakatta group using KN-93, myristoylated AIP, and W-7 to inhibit CaMKII (Yaniv et al., 2013) suggest that CaMKII may affect SAN automaticity by actions on metabolism. In our opinion, these results are intriguing but inconclusive because of the documented off-target actions of these reagents (Ledoux et al., 1999; Chatelier et al., 2005; Gao et al., 2006; Rezazadeh et al., 2006; Liao et al., 2011). Taken together, these studies support a view that CaMKII is not required to maintain basal heart rates but plays a critical role in sustained heart rate increases during physiological stress. This selective role of CaMKII on heart rate suggests that CaMKII inhibition could protect against excessive heart rates without reducing baseline heart rate.

## CaMKII IN SND

**FIGURE 2:**
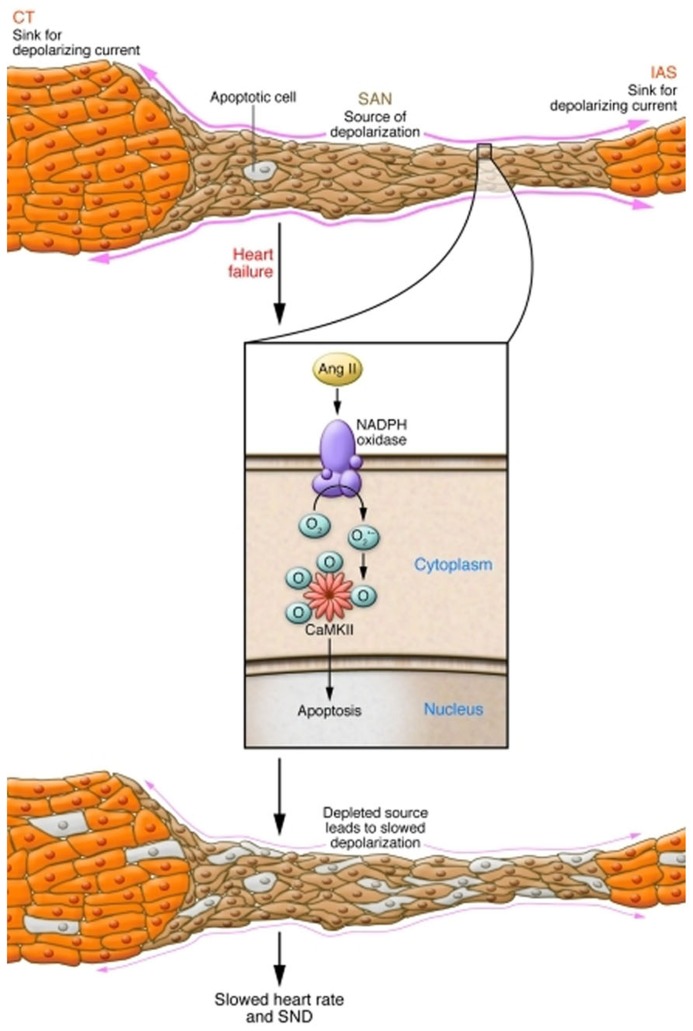
**Mechanism of Ang II-induced SND**. Normally, the small volume of excited tissue in the SAN (source) depolarizes the neighboring quiescent atrial tissue (sink). In conditions with increased Ang II, NADPH oxidase is activated, leading to oxidation of two methionine residues of CaMKII, rendering the enzyme autonomously active. Elevated activity of CaMKII leads to SAN cell death, reducing the threshold volume of automatic cells of the SAN and increasing non-excitable tissue in the form of fibrosis. This increased electrotonic loading produces a source-sink mismatch slows the beating rate, and causes SND. CT, crista terminalis; IAS, inferior atrial septum. Reproduced from Huke and Knollmann (2011), with permission from JCI.

Conditions that favor SND, such as heart failure, atrial fibrillation (AF), and advanced age are marked by heightened ROS (Cesselli et al., 2001; Kim et al., 2005; Dai et al., 2009). Because CaMKII is activated by ROS (Erickson et al., 2008) in the setting of increased angiotensin II (Ang II), a circulating neurohormone present at increased levels in heart failure, we tested if oxidized CaMKII (ox-CaMKII) could contribute to SND. We found Ang II increased atrial and SAN oxidation by activating NADPH oxidase, leading to increased ox-CaMKII, SAN cell apoptosis, and SND (Swaminathan et al., 2011). In order to test whether elevated ox-CaMKII could cause SND, mice were infused with Ang II. Ang II infusion for 3 weeks caused increased SAN ox-CaMKII, SAN cell apoptosis, fibrosis, slowed atrial impulse conduction velocity, and SND. Ang II-triggered SND was prevented by transgenic myocardial and SAN cell expression of AC3-I (Zhang et al., 2005) and by SAN-targeted gene therapy (Kikuchi et al., 2005) providing ectopic SAN expression of a CaMKII inhibitory peptide, CaMKIIN, that is endogenous to neurons but absent in heart (Chang et al., 1998). Neither transgenic nor gene-targeting approaches to SAN CaMKII inhibition affected the hypertensive response to Ang II, nor did they abrogate the increased SAN ROS due to Ang II infusion, indicating that CaMKII was a critical downstream signal for the pathological actions of ROS on SAN. The increase in SAN ox-CaMKII by Ang II required activation of NADPH oxidase, because it was absent in *p47*^-^^/^^-^ mice (Huang et al., 2000) lacking functional NADPH oxidase. We developed a structural and computational model of the SAN that revealed a quantitative mechanism to explain how Ang II-induced SAN cell apoptosis resulted in SND by reducing SAN cell number and increasing electrotonic loading of surviving SAN cells to cause loss of high-fidelity impulse formation and propagation (**Figure 2**; Huke and Knollmann, 2011). We also found that right atrial tissue from patients with heart failure who required artificial pacemakers for SND or dogs with pacing-induced heart failure and SND had elevated ox-CaMKII compared with patients with heart failure alone or dogs with non-SND controls. These findings provide insights into how excessive activation of CaMKII in SAN cells causes SND, suggest ox-CaMKII is a biomarker for SND and identify what we believe to be a novel candidate approach to preventing SND in high risk settings by CaMKII inhibition.

Patients with AF are at increased risk for SND (Chang et al., 2013) and CaMKII activity and expression are increased in fibrillating human atria (Neef et al., 2010). We recently found that ox-CaMKII is increased in fibrillating compared to non-fibrillating human atria and that Ang II infusion increases AF induction in mice (Purohit et al., 2013). Mice with transgenic expression of AC3-I, mice with a knock-in mutation (MM-VV) in CaMKIIδ that prevents oxidative activation and mice with transgenic over-expression of methionine sulfoxide reductase A that reverses the first oxidation state (sulfoxide) of methionine were all resistant to Ang II-induced AF. We interpret these findings to suggest that ox-CaMKII is a unifying signal for SND and AF.

Diabetes is a risk factor for SND (Podlaha and Falk, 1992). We recently found significantly more ox-CaMKII in right atrium from patients with a history of diabetes and myocardial infarction (MI) compared with right atrial tissue from patients with MI but no diabetes, suggesting that ox-CaMKII could contribute to the increased mortality in diabetic patients after MI (Luo et al., 2013). Streptozotocin (STZ)-treated mice develop severe type I diabetes due to death of pancreatic β-cells. STZ-treated diabetic mice were twice as likely to die after MI surgery as vehicle-treated control mice, mimicking the increased mortality in diabetic patients compared with that in non-diabetic patients after MI. STZ-treated MM-VV mice and mice with transgenic myocardial and SAN expression of AC3-I (Zhang et al., 2005) were protected from increased mortality after MI, indicating that increased ox-CaMKII was essential for excess mortality after MI in STZ-treated mice. Death in STZ-treated mice after MI was due to severe bradycardia, consistent with known defects in cardiac pacemaker function in another diabetic animal model (Howarth et al., 2007). In contrast to our earlier studies with Ang II-triggered ROS by activation of NADPH oxidase (Swaminathan et al., 2011), we found that hyperglycemia-induced ROS were primarily from mitochondria. Excess mortality in STZ-treated diabetic mice after MI surgery was prevented by chronic infusion with a mitochondrial targeted antioxidant, Mito-TEMPO. Mito-TEMPO reduced ox-CaMKII, preserved heart rates, and improved survival after MI. These results provide new evidence that ox-CaMKII is a biomarker for SND and suggest that mitochondrial or CaMKII-targeted antioxidant therapies could benefit high-risk diabetic patients.

In summary, CaMKII appears to play important roles in tuning the fight or flight response and in promoting SND. It may be that the physiological benefits of CaMKII activation in early life are outweighed in later life by the tendency of CaMKII to become persistently active under conditions of high oxidative, neurohumoral and hyperglycemic stress. The tractability of CaMKII as a target for selectively controlling heart rate and preventing SND will depend upon the availability of clinically suitable CaMKII inhibitory drugs or gene therapy.

## Conflict of Interest Statement

Mark E. Anderson is a cofounder of Allosteros Therapeutics, a biotech aiming to treat cardiovascular disease by enzyme inhibition.
